# Increased Expression of Vascular Endothelial Growth Factor-D Following Brain Injury

**DOI:** 10.3390/ijms20071594

**Published:** 2019-03-30

**Authors:** Sukriti Nag, Janet Manias, James H. Eubanks, Duncan J. Stewart

**Affiliations:** 1 Department of Laboratory Medicine and Pathobiology, University of Toronto, Toronto, ON M5S 3E8, Canada; jmanias@gmail.com; 2 Department of Pathology, Rush University Medical Center, Chicago, IL 60612, USA; 3 Department of Physiology, University of Toronto and Krembil Research Institute, University Health Network, Toronto, ON M5G 2A2, Canada; jeubanks@uhnres.utoronto.ca; 4 Department of Medicine, University of Ottawa, and Ottawa Hospital Research Institute, Ottawa, ON K1H 8L6, Canada; djstewart@ohri.ca

**Keywords:** angiogenesis, blood–brain barrier, brain Injury, brain trauma, cold-injury, VEGF-A, VEGF-B, VEGF-D, VEGFR-2, VEGFR-3

## Abstract

Alterations in the expression of the vascular endothelial growth factors (VEGF) A and B occur during blood–brain barrier (BBB) breakdown and angiogenesis following brain injury. In this study, the temporal and spatial expression of VEGF-D and VEGF receptors-2 and -3 (VEGFR-2 and VEGFR-3, respectively) was determined at the mRNA and protein level in the rat cortical cold-injury model over a period of 0.5 to 6 days post-injury. In order to relate endothelial VEGF-D protein expression with BBB breakdown, dual labeling immunofluorescence was performed using antibodies to VEGF-D and to fibronectin, a marker of BBB breakdown. In control rats, VEGF-D signal was only observed in scattered perivascular macrophages in the cerebral cortex. The upregulation of VEGF-D mRNA expression was observed in the injury site between days 0.5 to 4, coinciding with the period of BBB breakdown and angiogenesis. At the protein level, intracerebral vessels with BBB breakdown to fibronectin in the lesion on days 0.5 to 4 failed to show endothelial VEGF-D. Between days 0.5 to 6, an increased VEGF-D immunoreactivity was noted in the endothelium of pial vessels overlying the lesion site, in neutrophils, macrophages, and free endothelial cells within the lesion. The upregulation of VEGFR-2 and -3 mRNA and protein expression was observed early post-injury on day 0.5. Although there was concurrent expression of VEGF-A, VEGF-B, and VEGF-D post-injury, differences in their spatial expression during BBB breakdown and angiogenesis suggest that they have specific and separate roles in these processes.

## 1. Introduction

Angiogenesis, a crucial component of tissue repair after brain injury, is modulated by growth factors, including members of the vascular endothelial growth factor (VEGF) family. Best characterized is VEGF-A, the first member of the family to be discovered. VEGF-A has biologic roles in vasculogenesis [1,2] and angiogenesis [3,4] and during increased vascular permeability [5,6] in noncerebral tissues. An Increased expression of VEGF-A during blood–brain barrier (BBB) breakdown and angiogenesis has been reported following brain injury [7,8,9], while fewer studies have documented an increased expression of VEGF-B following brain injury [9] and in systemic tumors [10]. VEGF-C and VEGF-D form another VEGF subfamily by virtue of the presence of the N- and C-terminal extensions that are not found in other VEGF family members [11,12]. Both VEGF-C and VEGF-D are synthesized and secreted as large precursor forms that are proteolytically processed by plasmin into mature forms comprising the central VEGF homology domain [13]. Mature forms of both VEGF-C and VEGF-D activate the transmembrane endothelial tyrosine kinase receptors—VEGF receptor-2 (VEGFR-2) and VEGF receptor-3 (VEGFR-3)—with a much higher affinity toward VEGFR-2, thus driving the growth of blood vessels and lymphatics [11,14]. 

A high VEGF-D mRNA expression was reported in murine fetal and adult lung [12,14,15,16] while in adult humans, VEGF-D transcript expression was strongest in the heart, lung, skeletal muscle, colon, and small intestine [11]. VEGF-D has been poorly studied since phenotypic changes were not observed following its knockout in mice [17,18]. However, since VEGF-D is a known endothelial cell mitogen [11], interest has centered on its role in angiogenesis and lymphangiogenesis in developing tissues and pathologies such as non-neural tumors [19,20,21]. Although an increased expression of VEGF-D protein was reported in human glioblastoma multiforme, a highly malignant brain tumor [22,23], there are no reports of VEGF-D expression in the adult brain in steady states and following non-neoplastic conditions such as brain injury.

In this study, the temporal and spatial localization of VEGF-D was detected at the mRNA level by quantitative reverse transcriptase polymerase chain reaction (qRT-PCR) and at the protein level by immunostaining over a time course of days 0.5 to 6 post-injury. Since mature VEGF-D preferentially binds to VEGFR-2 and VEGFR-3, the expression of both these receptors at the mRNA and protein level was also detected post-injury. Endothelial VEGF-D protein expression was related to BBB breakdown by dual labeling immunofluorescence using antibodies to VEGF-D and to fibronectin, a marker of BBB breakdown. Microvessel density at the lesion margins at different time points post-injury was quantitated to assess the degree of angiogenesis. In addition, the cell types in the lesion margin at the different time points post-injury were quantitated, and the percentage of these cells showing VEGF-D immunoreactivity was documented.

In order to study the association of VEGF-D with BBB breakdown, a model was required, in which BBB breakdown occurred in every rat in the group and in which angiogenesis was also a finding in the model. These criteria were met by the rat cortical cold-injury model which was developed by Igor Klatzo [24] to study the pathophysiology of vasogenic edema which is a known complication of many types of brain injury such as trauma, tumors, hemorrhages, infarcts, and infections [25]. Less well-recognized is the finding that florid angiogenesis occurs in the lesion site, making this model suitable for in vivo studies of cerebral angiogenesis as documented previously [26,27,28,29,30]. The use of this model allowed us to compare the VEGF-D expression with that of VEGF-A and -B [9], which was also documented using this model.

## 2. Results

### 2.1. Morphology of the Cortical Cold Injury

**Figure 1:**
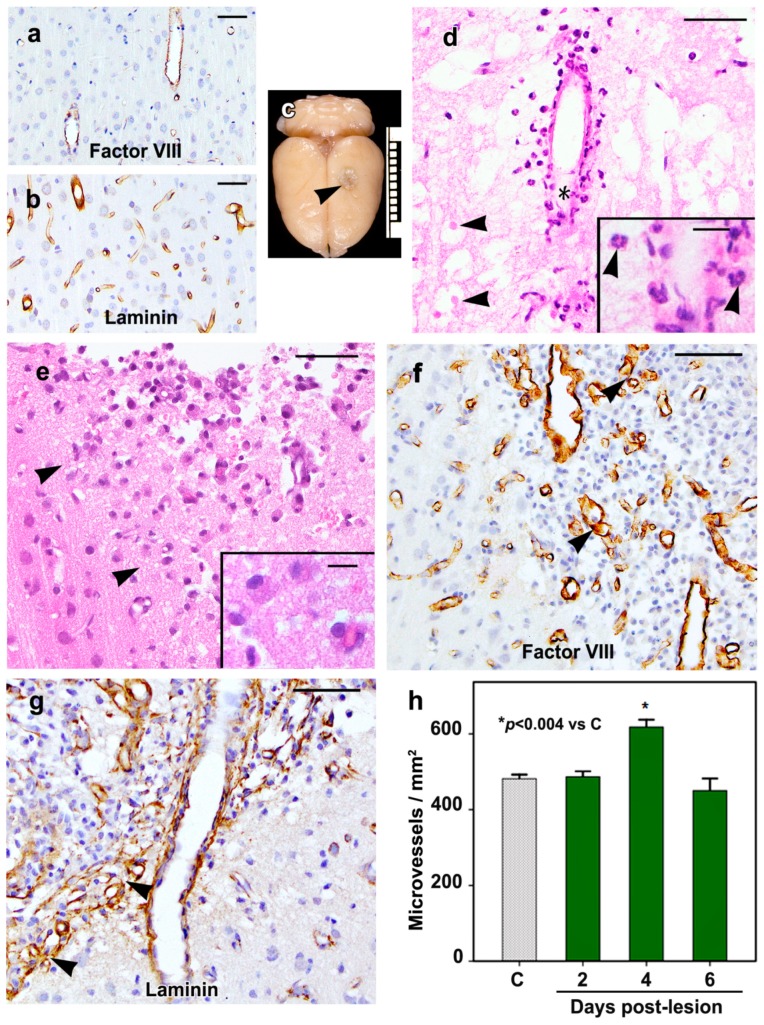
The findings in the control (**a,b**) and cold-injured rat cortices (**c–h**) are shown. The cortices of the control rats show factor VIII (**a**) and laminin (**b**) immunostaining in vessels. (**c**) The rat brain shows a day-4 lesion (arrowhead) which is circular at the brain surface. The scale represents 1 cm. The morphologies of the cortical cold-injury on days 0.5 (**d**) and 2 (**e**) are shown in hematoxylin-eosin stained sections. (**d**) The lesion area shows necrosis of the neuropil with degenerating neurons (arrowheads) and neutrophil infiltrates around a surviving vessel at the lesion margin. The lower part of the vessel marked with an asterisk is shown at a high magnification in the inset which shows the characteristic multilobed nuclei of neutrophils. (**e**) The margin of a day-2 lesion is marked by black arrowheads on the left side of the photomicrograph. Note the macrophage infiltration in the lesion area. The inset shows a higher magnification of 4 macrophages with oval eccentric nuclei. The margins of day-4 lesions show endothelial factor VIII (**f**) and laminin (**g**) immunoreactivity in the lesion and perilesion vessels. (**f**) Fewer microvessels show factor VIII (arrowheads) than laminin immunostaining (arrowheads, **g**). (**h**) The quantitation of microvessels at the lesion margin shows a significant increase (*p* < 0.004) in the microvessels on day 4 post-lesion. The bars represent mean ± SEM. *n* = 6 rats/group. Scale bars = 50 µm and inset scale bars = 10 µm.

The control rat brains showed no morphological changes, including the cortex underlying the sham-operated site. Immunostaining for factor VIII (Figure 1a) and laminin (Figure 1b) showed greater microvessel labeling with the anti-laminin antibody as compared to the anti-factor VIII antibody; therefore, the quantitation of the microvessel density at the lesion margin was done in sections immunostained for laminin. 

At the brain surface, the cold lesion was circular (Figure 1c), having a mean diameter of 2.1 (S.D. ± 0.3) mm and extended into cortical layer 4 as a shallow bowl-shaped lesion. Morphological changes in the cortical cold lesions were similar to our previous observations, including the time course of BBB breakdown, angiogenesis, and the inflammatory cell responses [9,27,29,30,31]. On day 0.5, the lesion area showed coagulative necrosis of the neuropil associated with neuronal loss and residual neurons in this area showed varying degrees of degeneration (Figure 1d). Few surviving vessels were noted in the subarachnoid space overlying the lesion site and within the lesion particularly at the margins. On day 0.5, neutrophils were noted in the lesion mainly in a perivascular location. Neutrophils had spherical outlines and multilobed nuclei (Figure 1d, inset). Neutrophil numbers decreased on day 2, and only rare neutrophils were seen on days 4 or 6. Macrophages noted on day 2 were larger than neutrophils and had eccentric oval- to bean-shaped nuclei (Figure 1e, inset). The macrophage numbers were maximal on day 4 and decreased on day 6.

On day 2, an increase in the number of free endothelial cells around preexisting vessels at the lesion margins was noted, and on day 4, these cells were scattered in the entire lesion area. These cells were polygonal in shape and had central nuclei, and they were immature since they failed to show factor VIII or laminin immunoreactivity (Figure 1f,g). On days 4 and 6, neovessels were interspersed among the free endothelial cells, particularly at the lesion margins (Figure 1f,g). The neovessels were clusters of thin-walled vessels having varying diameters due to microvascular remodeling, and their mean diameter was 5.82 (S.D. ± 1.83) µm. The quantitation of microvessels at the lesion margin showed a mean of 482 ± 11 and 487 ± 14 microvessels/mm^2^ in control rat brains and on day 2 post-lesion, respectively (Figure 1h). The microvessels were significantly increased on day 4 (*p* < 0.004), their mean value being 618 ± 19 microvessels/mm^2^, while on day 6, the mean number of microvessels was 450 ± 32/mm^2^, a value which is not significantly different from that of the mean microvessels in the cerebral cortex of control rats.

### 2.2. VEGF-D mRNA Expression

**Figure 2:**
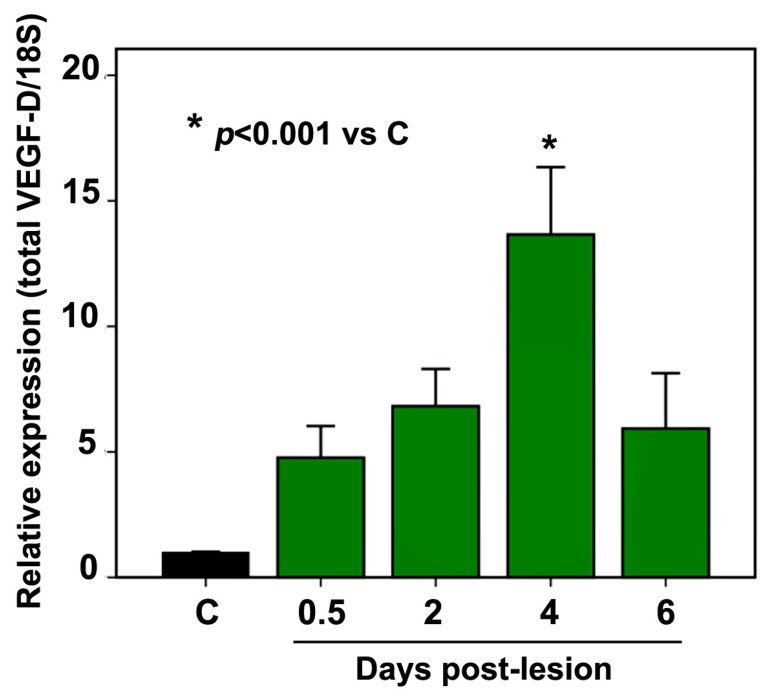
Quantitative RT-PCR analyses of total VEGF-D mRNA levels relative to 18S ribosomal RNA of control and cold-injured rats on days 0.5, 2, 4 and 6 post-lesion are shown. The mRNA expression in the brains of the cold-injured rats is increased as compared to that of the control rats starting on day 0.5 and is maximal on day 4 (*p* < 0.001) post-lesion, being almost 14-fold greater than the values of the control rat brains. The bars represent mean ± SEM. *n* = 6 rats/group.

VEGF-D mRNA expression was increased at the cold-injury site as compared to the corresponding cortices of the control rats, the increase being about 5-fold and 7-fold on days 0.5 and 2, respectively (Figure 2). A maximal increase in VEGF-D mRNA expression occurred on day 4 when about a 14-fold increase (*p* < 0.001) was observed. On day 6, the VEGF-D mRNA levels were still elevated, although the change was not statistically different (*p* = 0.322) from the values observed in the control rats.

### 2.3. VEGF-D Protein Expression in Control Rats

**Figure 3:**
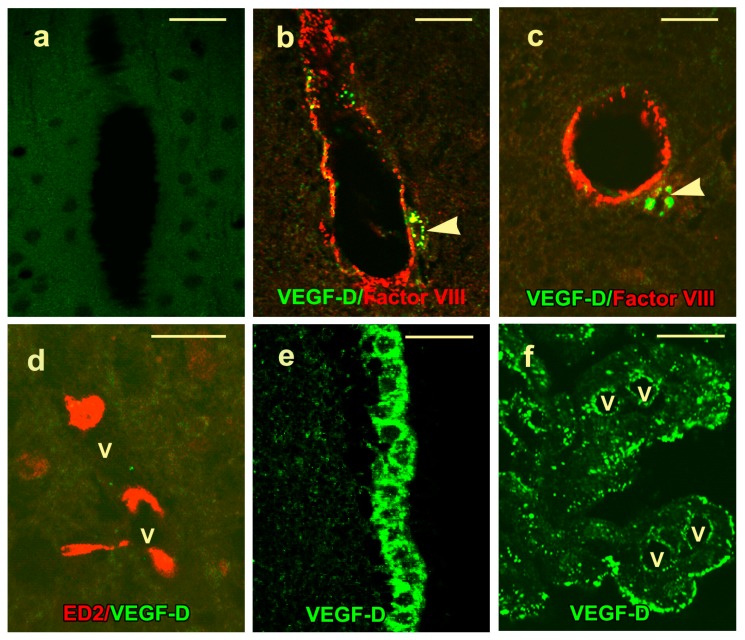
Localization of VEGF-D protein in the control rat brains (**a–f**) is shown. (**a**) No signal is detectable in the cortex of a control rat reacted with nonimmune serum. Merged confocal images of the control rat cortices show factor VIII signal in endothelial cells lining a vein (**b**) and an arteriole (**c**). Note the VEGF-D signal in the perivascular macrophages (arrowheads). (**d**) A merged confocal image from a cryostat section shows an ED2 signal in perivascular macrophages adjacent to vessel lumina (V). These cells fail to show the colocalization of ED2 and VEGF-D. Single-channel confocal images show a dot-like VEGF-D positivity in the ependymal cell cytoplasm (**e**) and in choroid plexus epithelial cells (**f**), particularly at their apical ends. Immunoreactivity is also present in the endothelial cells adjacent to the lumina of the stromal vessels that are marked with “V”. Scale bars = 25 µm.

No signal was noted in the cortex of control rats reacted with nonimmune serum (Figure 3a). Both the anti-VEGF-D antibodies utilized gave similar results, and the only VEGF-D signal observed in the cortex of control rats was cytoplasmic immunoreactivity in the perivascular macrophages of scattered venules and terminal arterioles (Figure 3b,c). These cells were interpreted to be perivascular macrophages since they were ED2 positive (Figure 3d) and nonreactive with antibodies against factor VIII, α-smooth muscle actin, or glial fibrillary acidic protein (GFAP). Dual labeling failed to show the colocalization of ED2 and VEGF-D (Figure 3d). VEGF-D protein was also observed in ependymal cells lining the ventricle (Figure 3e) in choroid plexus epithelium and endothelium (Figure 3f) and in the subfornicial organ. Both by immunohistochemistry and by immunofluorescence, the VEGF-D signal had a punctate quality.

### 2.4. VEGF-D Protein Expression During BBB Breakdown

**Figure 4:**
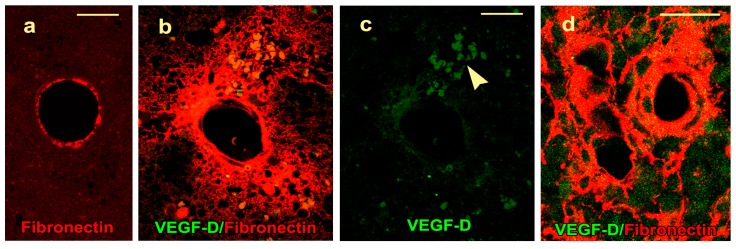
The control and lesion vessels during BBB breakdown are shown. (**a**) A single-channel confocal image shows a cortical arteriole of a control rat with fibronectin immunostaining of the basement membrane with no extravasation of fibronectin into the neuropil. (**b**) On day 0.5, a merged confocal image shows leakage of fibronectin through the vessel walls into the neuropil. (**c**) The green channel of the same image shows a lack of endothelial VEGF-D protein signal. Damage to the vessel wall has resulted in not only leakage of fibronectin but also a cluster of red blood cells (arrowhead) which are non-nucleated and of uniform size, and while few have circular outlines, others have a crenated outline. (**d**) On day 4, BBB breakdown involves an arteriole, veins, and neovessels; however, the endothelium of these vessels fails to show VEGF-D immunoreactivity. Scale bars = 25 µm.

The control rats showed fibronectin in the basement membrane of large caliber arterioles and the corresponding-sized veins in the cerebral cortex (Figure 4a). Blood–brain barrier breakdown, which is characterized by the extravasation of fibronectin through vessel walls into the surrounding neuropil, was not noted.

The lesion site of the cold-injured rats showed two phases of BBB breakdown to fibronectin. An initial phase on day 0.5 involved 10–14 arterioles and the corresponding-sized venules at the margins of the lesion (Figure 4b). These vessels failed to show any endothelial VEGF-D protein (Figure 4c). The second phase of the BBB breakdown accompanied angiogenesis starting at day 2; was maximal on day 4; and affected arterioles, venules, and neovessels at the lesion site (Figure 4d). The endothelium of these permeable vessels also failed to show colocalization of fibronectin and VEGF-D (Figure 4d).

### 2.5. Density of Inflammatory and Free Endothelial Cells and Their Numbers Expressing VEGF-D Protein

**Figure 5:**
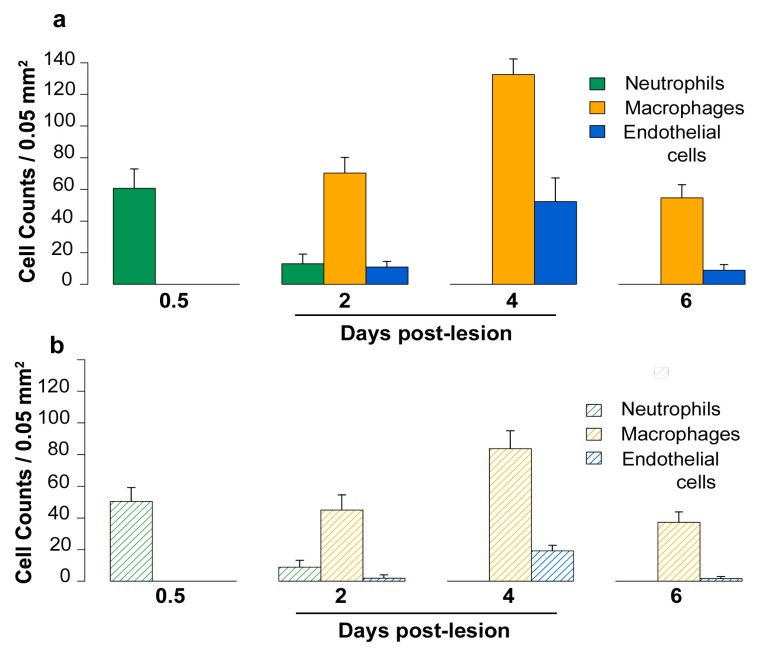
(**a**) The density of the total neutrophils, macrophages, and free endothelial cells/0.05 mm^2^ at the lesion margin on days 0.5, 2, 4, and 6 post-lesion is shown. Neutrophils were maximal on day 0.5, while macrophages and endothelial cells were maximal on day 4. (**b**) The hatched bars represent the mean numbers of neutrophils, macrophages, and free endothelial cells in the same fields, showing cytoplasmic VEGF-D immunoreactivity at the different time periods. The bars represent means ± SEM. *n* = 6 rats/group.

The distribution of inflammatory and free endothelial cells was not uniform throughout the lesion. Cell density appeared to be maximal at the lesion margin as compared to the center; therefore, quantitation of the total number of inflammatory and free endothelial cells/0.05 mm^2^ at the different time points and their numbers showing VEGF-D immunoreactivity was done at the lesion margins (Figure 5a,b). 

**Figure 6:**
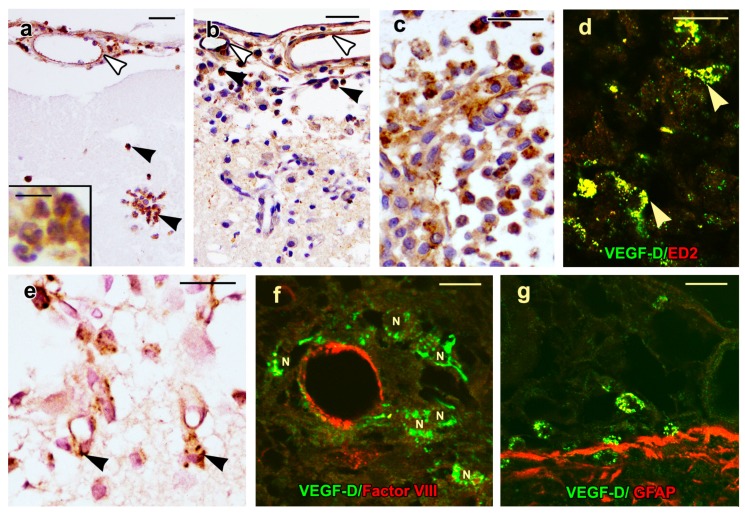
VEGF-D immunoreactivity in inflammatory and free endothelial cells is shown. (**a**) A light microscopy image of a day-0.5 lesion shows infiltrating neutrophils (arrowheads), with VEGF-D immunoreactivity. The inset shows a higher magnification of the neutrophils with their characteristic multilobed nuclei. (**b**) A day-2 lesion shows macrophages, some of which show VEGF-D protein (black arrowheads). The endothelial cells lining the pial vessels show VEGF-D signal on days 0.5 (**a**) and 2 (**b**) post-injury (white arrowheads). (**c**) On day 4, the increased numbers of macrophages in the lesion show marked cytoplasmic VEGF-D immunostaining by light microscopy. (**d**) On day 4, the merged confocal image shows perivascular macrophages (arrowheads) with colocalization of ED2 and VEGF-D. (**e**) On day 4, cytoplasmic VEGF-D positivity is present in the polygonal-shaped endothelial cells (arrowheads) adjacent to neovessels. (**f**) On day 4, the merged confocal image shows cytoplasmic, granular VEGF-D immunoreactivity in endothelial cells which are migrating from a preexisting vessel with endothelial Factor VIII positivity (red signal). The nuclei of the free endothelial cells are labeled with “N”. (**g**) On day 6, cytoplasmic VEGF-D positivity is present in macrophages in the subarachnoid space, while astrocytic processes in the glia limitans show only glial fibrillary acidic protein (GFAP; red signal) with no evidence of the colocalization of VEGF-D and GFAP. Scale bars = 25 µm and inset scale bar = 10 µm.

Three to five pial vessels overlying the lesion site were morphologically intact post-lesion, and on days 0.5 to 6, endothelial cells lining these vessels showed VEGF-D immunoreactivity (Figure 6a,b). On day 0.5, a mean of 61 (± 6) neutrophils/0.05 mm^2^ were present at the lesion margin, and 83% of these cells showed cytoplasmic VEGF-D immunostaining (Figure 6a, inset). On day 2, the mean number of neutrophils decreased to 13 (± 3)/0.05 mm^2^; however, 69% of neutrophils still showed VEGF-D immunoreactivity. On days 2, 4, and 6, VEGF-D was present in two types of mononuclear cells at the lesion margin. The first type was a macrophage which was first noted on day 2 (Figure 6b), its mean number being 70 (± 5)/0.05 mm^2^, while their numbers were maximal on day 4 (Figure 6c), the mean being 133 (± 5)/0.05 mm^2^. The percentage of macrophages showing cytoplasmic VEGF-D immunostaining was similar on days 2 and 4, being 64% and 63%, respectively. On day 4, perivascular macrophages showed colocalization of ED2 and VEGF-D (Figure 6d). The other type of mononuclear cell showing VEGF-D positivity was larger, had a polygonal shape with central nuclei, and represented free endothelial cells (Figure 6e,f). The mean values of their total numbers were 11 ± 2 on day 2 and 52 ± 7/0.05 mm^2^ on day 4 while their percentages showing cytoplasmic VEGF-D labeling were 18% and 37% on days 2, and 4, respectively. Although these cells were nonreactive with the factor VIII antibody (Figure 1f), they failed to show α-smooth muscle actin, indicating that they were not pericytes. 

On day 6, macrophage (Figure 6g) and endothelial cell numbers were reduced, their means being 55 (± 4) and 9 (± 2)/0.05 mm^2^, respectively. At this time period, 68% of macrophages and 19% of endothelial cells still showed VEGF-D immunoreactivity. The VEGF-D protein was not observed in other cell types, such as astrocytes (Figure 6g).

### 2.6. VEGFR-2 and VEGFR-3 mRNA and Protein Expression

**Figure 7:**
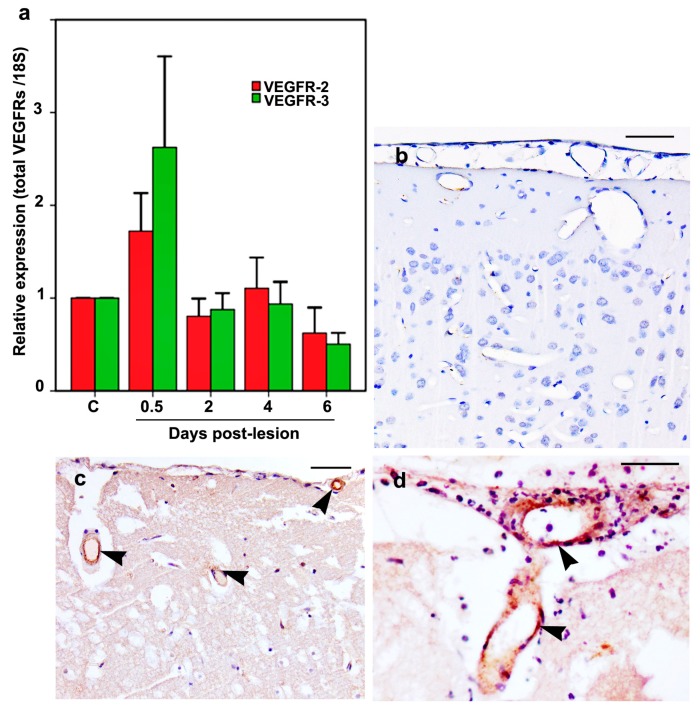
The VEGFR-2 and VEGFR-3 mRNA (**a**) and protein (**b**–**d**) expression are shown. (**a**) Quantitative RT-PCR analyses of the total VEGFR-2 (red bars) and VEGFR-3 (green bars) mRNA relative to 18S of the control and cold-injured rats on days 0.5, 2, 4, and 6 post-lesion are shown. The VEGFR-2 and VEGFR-3 mRNA expression is increased in the cold-injured rats as compared to control rats, on day 0.5 post-lesion. The bars represent mean ± SEM. For each receptor and the control rat groups, *n* = 6 rats/group. (**b**) The cortex of a control rat shows the lack of a signal for VEGFR-2 on day 0.5 by immunohistochemistry. (**c**) On day 0.5, 3 vessels (arrowheads) show increased endothelial immunoreactivity for VEGFR-2. (**d**) On day 0.5, marked endothelial immunoreactivity (arrowheads) for VEGFR-3 protein is present in a pial and intracerebral vessel. Scale bars = 50 µm.

Although both VEGFR-2 and VEGFR-3 mRNA expression in the cold-injured rats was increased by about 2-fold and 3-fold, respectively, on day 0.5 as compared to the mRNA of the control rats, their values did not reach statistical significance (Figure 7a). At the subsequent time points, their mRNA values were also similar to that of the control rats.

VEGFR-2 and VEGFR-3 proteins were not detected in the endothelium of pial or the intracerebral vessels of the control rats (Figure 7b). The cold-injured rats showed endothelial immunoreactivity for VEGFR-2 (Figure 7c) and VEGFR-3 (Figure 7d) on days 0.5 and 2 post-lesion, in the 3–5 pial vessels overlying the lesion and a similar number of intracerebral vessels per lesion. The number of pial and intracerebral vessels with endothelial immunoreactivity for VEGFR-2 and VEGFR-3 decreased on day 4, while on day 6, endothelial immunoreactivity for VEGFR-2 was present in only rare vessels while endothelial VEGFR-3 immunoreactivity was no longer observed. 

## 3. Discussion

To the best of our knowledge, this is the first study reporting increased expression of VEGF-D mRNA and protein following brain injury. The time course of increased VEGF-D transcript and protein expression coincided with the period of BBB breakdown and angiogenesis following brain injury. While VEGF-D expression is related to angiogenesis, its expression is unrelated to the phases of BBB breakdown post-injury.

The cold-injury model has been utilized extensively to study BBB breakdown, the initiating factor in vasogenic edema using tracers such as Evans blue [32] and horseradish peroxidase (HRP) [27,30] and also by the immunolocalization of extravasated circulating proteins such as serum proteins [27], fibrinogen [33], and fibronectin [8,29,30] through vessels into the surrounding brain. Since all vessels within the lesion do not show BBB breakdown, immunofluorescence allows the identification of vessels leaking proteins while dual labeling allows the localization of a protein of interest in the endothelium of vessels leaking proteins in the same animal. Using these techniques, two phases of BBB breakdown have been observed after brain injury, an early phase (day 0.5) during which there is an extravasation of serum proteins from the lesion site into the underlying white matter that spreads via the corpus callosum into the white matter of the contralateral hemisphere [27]. This early phase of BBB breakdown is deleterious to the patient since it leads to vasogenic edema, which, if extensive, can lead to brain herniation and death [25]. The other phase of BBB breakdown occurs during angiogenesis (days 2–4), and this phase promotes healing since it lays down a matrix which facilitates the migration of cells within the lesion and ingrowth of vessels [28,34]. The time course of BBB breakdown in the present study is similar to our previous observations; however, lesion vessels showing BBB breakdown on days 0.5 to 4 failed to show any endothelial VEGF-D protein. In this respect, VEGF-D is similar to VEGF-B, which, in our previous study, failed to show any endothelial VEGF-B protein in vessels leaking protein [9], suggesting that both VEGF-B and VEGF-D are unrelated to vascular hyperpermeability. In contrast, VEGF-A, a known inducer of vascular hyperpermeability, is expressed in the endothelium of vessels leaking proteins [8,9].

VEGF-D was discovered as a c-fos-induced mitogen [35]. In this respect, it is pertinent that increased c-fos expression was demonstrated in the lesion and perilesion areas in this model between days 0.5 and 6 post-injury [27]. The time course of angiogensis, in this study, is similar to our previous observations in the cold-injury model [27,28]. An increase in the number of free endothelial cells around preexisting vessels was observed on day 2, while on day 4, these cells were scattered throughout the lesion site. The increased cell numbers is due to endothelial proliferation which, by bromodeoxyuridine labeling, is maximal between days 2 and 4 [9,28]. These free endothelial cells are immature, since they fail to show factor VIII and are not immunoreactive with endothelial markers such as glucose transporter-1 [27] or endothelial nitric oxide synthase [31] until they mature and line vessel lumina. However, these cells do show cytoplasmic expression of occludin [29] and VEGF-A and B protein expression [9] and, in this study, show cytoplasmic VEGF-D protein as well. 

In this study, neovessel formation was maximal on day 4, accounting for the significant increase in microvessel density, and coincided with the period of maximal VEGF-D mRNA expression. The values of the microvessel density on day 4 may be an underestimate since scattered microvessels were dilated; therefore, potentially fewer vessels would be present in the defined areas. Although VEGF-D is known to induce lymphatics in murine tumor models [21,36], the microvessels observed at the lesion margins in this study do not represent lymphatics, which, in the CNS, are located adjacent to dural sinuses [37] and, therefore, are anatomically distant from the cold-injury site. Our previous studies have also reported an increased expression of both VEGF-A and VEGF-B mRNA at the lesion site during the period of maximal endothelial proliferation with VEGF-A mRNA levels peaking on days 3 and 4 post-injury, while VEGF-B mRNA was increased between days 2 and 6 [9]. 

At the protein level, increased VEGF-D immunoreactivity was observed in endothelia of non-leaking pial vessels, a feature similar to VEGF-A localization, while VEGF-B, which is constitutively expressed in endothelial cells of all cerebral vessels, showed decreased endothelial expression of this protein post-injury with recovery of the endothelial VEGF-B protein expression on day 6 [9]. A comparison of the angiogenesis-inducing properties of different human VEGFs demonstrates that both VEGF-A and D show similar angiogenic activity when transferred locally to the periadventitial space of rabbit carotid arteries [38]; however, VEGF-D is the strongest inducer of angiogenic activity upon adenoviral transfection of rabbit skeletal muscles [39]. 

VEGFR-2 [40] and VEGFR-3 [41] are widely expressed during early embryonic endothelial cell development. Later in the fetus, VEGFR-2 is expressed in vascular endothelial cells [40] while VEGFR-3 becomes confined to lymphatic endothelial cells [41]. However, signaling from either VEGFR-2 or VEGFR-3 can modulate both blood vessels or lymphatics [42,43]. In adult vessels, these receptors are downregulated but reinduced during transient phases of physiological angiogenesis and a variety of pathological conditions [1,23]. In this study, increased expression of both VEGFR-2 and VEGFR-3 transcripts was observed on day 0.5, although values failed to reach statistical significance, possibly due to the small group size. Also, since the brain endothelial volume is <0.1% of the total brain volume [44], analyses using total mRNA may underestimate the increase of endothelial VEGFR mRNA expression. In addition to the increased expression of VEGF-D in this model, another factor accounting for the increased VEGFR-2 expression is the concurrent increased expression of VEGF-A [9], which is a major ligand for this receptor. Other studies have demonstrated increased VEGFR-2 mRNA expression by in situ hybridization in brain tumors [45] and brain contusions [46] but not in the adjacent normal brain. 

The brains of the control rats failed to show VEGFR-2 or VEGFR-3 proteins, supporting previous studies which reported a lack of immunoreactivity for VEGFR-2 [46,47,48] and VEGFR-3 [23] proteins in normal brain. In this study, increased endothelial expression of both VEGFR-2 and VEGFR-3 proteins was observed on days 0.5 and 2 post-injury, during the period when the increased VEGF-D expression occurs. Other studies have documented an increased expression of VEGFR-2 protein in the early phase after brain contusion in rat [46], following the administration of VEGF-A in an adult rat brain [47] and following a 6-min exposure of an adult rat cortice to an ultraviolet beam [48]. In the case of VEGFR-3, increased endothelial immunoreactivity was reported in glioblastomas [23].

Marked cytoplasmic VEGF-D immunoreactivity was observed in neutrophils and macrophages. This is unlikely to be due to the endogenous peroxidase content of neutrophils since the cytoplasmic localization of VEGF-A and VEGF-B has also been reported in neutrophils by both the immunoperoxidase [9] and immunofluorescence methods [9,49]. Several types of macrophages are present in the injury site between days 2 to 6. A proportion of these macrophages are derived from resident microglia since they show OX42 positivity as reported previously [9]. In this study another population of macrophages—the perivascular macrophages—were identified using the ED2 antibody, a known marker of the perivascular macrophages [50,51,52]. The perivascular macrophages of the control rats failed to show colocalization of VEGF-D and ED2, possibly due to the low VEGF-D signal in steady states, while strong colocalization was observed in the activated perivascular macrophages within the lesion. Astrocytes within the lesion or in the perilesional area did not show VEGF-D immunoreactivity, although neoplastic astrocytes in glioblastoma are known to express VEGF-D protein [22]. In this respect, VEGF-D differs from VEGF-A and VEGF-B proteins, which are expressed in astrocytes post-injury. 

The high expression of VEGF-D during angiogenesis may explain why the inhibition of VEGF-A alone is ineffective in treating neoplastic angiogenesis and why the benefit, even for responding patients, is usually modest [53]. Anti-VEGF-A therapy alone may also be ineffective since the inhibition of VEGF-A results in an upregulation of VEGF-D [54]. The therapeutic potential of the administration of VEGF-D by a viral gene transfer is being studied in nonneoplastic systemic conditions with promising results [55]. While clinical trials have not extended to brain diseases, antiangiogenesis therapy for the treatment of brain tumors may be more effective if a combination of both VEGF-A and -D inhibitors are used or if inhibitors of VEGFR-2 are used, as this receptor is activated by both VEGF-A and VEGF-D. In brain trauma, the administration of VEGF-D alone post-injury may be beneficial in promoting repair due to its strong angiogenic and neural effects, since it is reported to be essential in the maintenance of the integrity of hippocampal neurons [56]. An added advantage of the administration of VEGF-D alone is that it lacks the potent vascular permeability enhancing property of VEGF-A which leads to brain edema and subsequent brain herniation and death in large hemispheric brain lesions such as infarcts and trauma

## 4. Materials and Methods

The protocol (AUP 144.7) involving the use of rats was in compliance with the guidelines set by the Canadian Council on Animal Care and was further approved by the University Health Network Animal Care Committee, Toronto, Canada. 

### 4.1. Cortical Cold-Injury Model

Cold injury was produced in male Wister rats (180–200 g) as described previously [30]. The rats were anesthetized by isoflurane inhalation (3% for induction and 2% for maintenance). A craniotomy was performed in the left parietal bone using a dental drill with a bit measuring 2.3 mm in diameter. A cold probe consisting of the tip of a copper wire, contained in a 20-mL syringe containing liquid nitrogen, was placed over the brain for 45 s. The craniotomy site was covered with gel foam, and the skin incision was approximated with metal clips. In the sham-operated control rats, all surgical procedures were carried out except for the production of the cold lesion. For all experiments, the data was collected from 6 cold-injured rat brains/group on days 0.5, 2, 4, and 6 along with 6 control rat brains/group.

### 4.2. RNA Preparation and qRT-PCR

The cold-injured and control rats were euthanized by decapitation; their brains were chilled on an ice-cold dissection disc, and the lesion areas and corresponding brain areas of the control rats were rapidly dissected and frozen on dry ice. The total RNA was extracted from the brain samples using TRIzol^®^ Reagent (Invitrogen Corp., Burlington, ON, Canada). The elimination of genomic DNA and reverse transcription using 1 μg of total RNA was performed using the Quantitech Reverse Transcription Kit (Qiagen, Toronto, ON, Canada. qRT-PCR was performed using the SYBR® Green PCR Master Mix (Applied Biosystems Inc., Foster City, CA, USA) on an ABI PRISM 7900HT Sequence Detection System (Applied Biosystems Inc.). All analyses were done in triplicate, and the data was normalized using the housekeeping gene 18S ribosomal RNA (18S) by calculating the difference in the cycle thresholds (ΔC_T_). The mRNA levels at each time period was compared with the mRNA of the controls by calculating the difference in Δ*C*_T_ (ΔΔ*C*_T_). The mRNA levels were then expressed as fold increases (2^−^^ΔΔ^^*C*T^) as per the double delta C_T_ method [57].

The VEGF-D primers were designed using Primer Express^®^ (Applied Biosystems Inc.), while the VEGFR-2 and VEGFR-3 primers were similar to those used in a previous study [58], as was the 18S primers [59]. The sequences for each primer pair were as follows: VEGF-D (sense: ACACCGAGCAGTGAAGGATG and antisense: CGCGGATCTGTTGTTCAGAA); VEGFR-2 (sense: TGGAGGATGTGGGCTATGAG and antisense: CAAGCAACCTTCCAAAACCA); VEGFR-3 (sense: AGCCAGATATTACAACTGTGTGTCC and antisense: GTCTATGCCTGCTTTCTATCTGCTC); and 18S (sense: GACGATCAGATACCGTCGTAGTTC and antisense: GTTTCAGCTTTGCAACCATACTCC).

### 4.3. Histological Analyses and Immunohistochemistry

The rats were euthanized by isoflurane inhalation and perfused with 3% paraformaldehyde in a 0.1 M phosphate buffer. Coronal slabs of brain containing the cold-injury site were processed for paraffin sectioning using standard techniques. Coronal sections (6 µm) of the cortex were stained with hematoxylin and eosin for histological analysis, and the adjacent sections were used for immunostaining. The brains of 6 additional day-4 test rats were frozen after fixation along with the brains of 6 control rats, and 10-µm cryostat sections were used for ED2 localization by immunofluorescence as described below.

Immunohistochemistry for the detection of VEGF-D, VEGFR-2, VEGFR-3 and laminin was performed by the indirect streptavidin-biotin peroxidase method [9]. The paraffin sections were pretreated with 0.5% pepsin in 0.01 M HCl for 30 min at 37 °C, followed by incubation in primary antibody overnight at 4 °C for VEGF-D and at room temperature for 2 hours for VEGFR-2 and -3 and laminin. The dilutions of antibodies used and their source were as follows: (1) polyclonal goat anti-VEGF-D antibody raised against a peptide mapping at the N-terminus of VEGF-D of mouse origin (1:20, Santa Cruz Biotechnology, Santa Cruz, CA, USA), (2) monoclonal mouse Flk-1/VEGFR-2 antibody (1:10, Santa Cruz Biotechnology); (3) polyclonal rabbit Flt-4/VEGFR-3 antibody (1:500, Santa Cruz Biotechnology), and (4) polyclonal rabbit anti-laminin antibody (1:150, Sigma-Aldrich, St Louis, MO, USA). Diaminobenzidine was used as a substrate for these reactions. The controls included the omission of primary antibodies and the replacement of the primary antibody with normal serum from the appropriate species. 

### 4.4. Immunofluorescence

Single- and dual-labeling immunofluorescence was done as described previously [60]. In addition to the VEGF-D antibody mentioned in Section 4.3., a polyclonal rabbit anti-VEGF-D antibody raised against amino acids 158–202 of VEGF-D of human origin (1:150, Santa Cruz Biotechnology) was used. The molecular weight of VEGF-D detected by both antibodies was 21 kDa (Figure S1). Dual labeling was done using paraffin sections to detect the colocalization of VEGF-D with (1) factor VIII (polyclonal anti-Factor VIII antibody, 1:750, Dako Canada Inc., Mississauga, ON, Canada); (2) fibronectin (polyclonal anti-fibronectin antibody, 1:150, Sigma Chem Co, St Louis, MO, USA); (3) glial fibrillary acidic protein (GFAP) (polyclonal anti-GFAP antibody, 1:3000, Dako Canada Inc); and (4) α-smooth muscle actin (polyclonal anti-α-smooth muscle actin antibody 1:400, ABCAM, Cambridge, MA, USA). The anti-fibronectin antibody reacted with both cellular and circulating fibronectin. Cryostat sections were used for the colocalization of VEGF-D and the ED2 (monoclonal ED2 (CD163) antibody, 1:80, Bio-Rad Labs Inc., Hercules, CA, USA). VEGF-D was first localized by an overnight incubation with the anti-VEGF-D antibody at 4 °C and then with the biotinylated rabbit anti-goat antibody (Jackson ImmunoResearch Laboratories Inc., Westgrove, PA, USA) for 30 min at room temperature followed by streptavidin-Alexa Fluor^TM^488 (Molecular Probes Inc., Eugene, OR, USA). In the case of the rabbit anti-VEGF-D antibody, the sections were treated with biotinylated goat anti-rabbit antibody for 30 min at room temperature, followed by streptavidin HRP and, finally, Tyramide Alexa Fluor ^R^488 (In Vitrogen Inc. Burlington, ON, Canada) as described previously [61]. The second protein was then detected by incubating the sections in the primary antibody at room temperature for 2 h followed by the goat anti-rabbit-Cy^TM^3 antibody (Jackson ImmunoResearch Laboratories Inc.). The sections were analyzed at 200× and 630× magnifications using a Zeiss LSM 510 META confocal laser-scanning microscope (Carl Zeiss Canada Ltd., Toronto, ON, Canada). The images were merged and converted to .tif images using the National Institutes of Health, Image-J software (http://www.rsb.info.nih.gov/ij) and Adobe Creative Suite 5 software (Adobe Systems Inc. San Jose, CA, USA).

### 4.5. Vessel and Cell Counts

The density of microvessels at the lesion margin and in the corresponding brain areas of the control rats was quantitated on days 0.5, 2, 4, and 6 as described previously [62]. Coronal sections (6 µm) were immunostained for laminin, a glycoprotein which is a major component of vessel basement membranes. Laminin was selected over the endothelial marker, factor VIII, since more microvessels were labeled with laminin than with factor VIII antibodies (Figure 1a,b) and since endothelial cells lining vascular tubes did not label with factor VIII. A rectangular tool of the MCID^®^ image analysis system was used to define the area for vessel counts at 3 points along the margin of the lesion and in corresponding areas of the control rat brains. Terminal arterioles/metaarterioles identified as vessels having a continuous or discontinuous smooth muscle layer and having diameters greater than 10 µm were not included in the measurement areas and were not included in the counts if present in the defined areas. Microvessels which included neovessels, capillaries, and postcapillary venules with vessel lumina and having diameters less than 10 µm in these defined areas were counted at a magnification of 200× to obtain the mean number of microvessels per mm^2^/group. 

The density of neutrophils, macrophages, and free endothelial cells/0.05 mm^2^ of the lesion margin on days 0.5, 2, 4, and 6 and their numbers showing VEGF-D immunoreactivity were quantitated using the Image-J cell counter software. Immunoreactivity was graded as positive or negative in three digital images taken along the lesion margin/rat at a magnification of 400×. The results were expressed as the mean number of cells/0.05 mm^2^. The cell types have been described in Section 2.1 and in our previous publications [9,61].

All counts were done in a blinded manner.

### 4.6. Statistical Analysis

The data are presented as mean ± SEM. The statistical significance in mRNA expression and vessel density between control and test groups was determined by the one-way analysis of variance followed by the Tukey HSD post hoc test. The data analyses were performed using the IBM SPSS Statistics 22 software package (Chicago, IL, USA). Significant differences were ascribed to *p* values < 0.05. 

## 5. Conclusions

**Figure 8:**
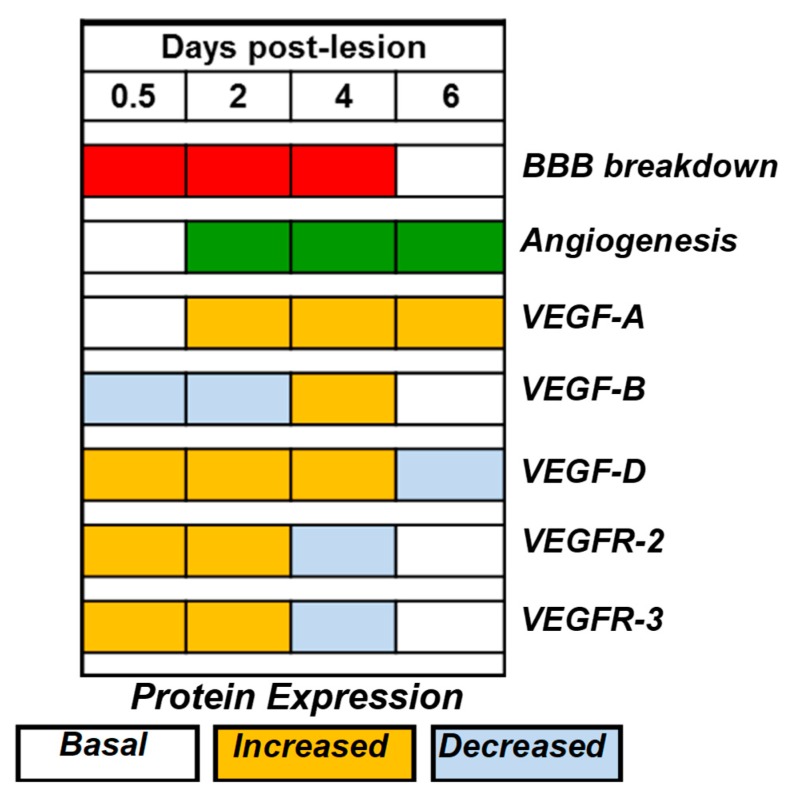
A diagram showing the concurrent temporal expression of VEGF-A, VEGF-B, and VEGF-D and the VEGFR-2 and VEGFR-3 proteins on days 0.5, 2, 4, and 6 post-injury during the period of BBB breakdown (red bars) and angiogenesis (green bars). The proteins were detected by immunohistochemistry and graded as basal, increased, or decreased immunoreactivity based on the VEGF expression in pial vessel endothelium, neutrophils, macrophages, and free endothelial cells. The data for the VEGF-A and B proteins are derived from a previous publication [25].

Our studies of the cold-injury model demonstrate the concurrent expression of VEGF-A, VEGF-B, and VEGF-D post-injury with minor differences in their temporal expression and more remarkable differences in their spatial expression at the protein level (Figure 8). While the increased expression of VEGF-A, VEGF-B, and VEGF-D is related to angiogenesis, the expression of VEGF-B and VEGF-D, unlike that of VEGF-A, is unrelated to the phases of BBB breakdown post-injury. Detailed studies of the temporal and spatial expression of growth factors are essential to determine effective therapeutic approaches to modulate the expression of these factors in the treatment of brain diseases.

## Supplementary Materials

Supplementary materials can be found at https://www.mdpi.com/1422-0067/20/7/1594/s1.

## Abbreviations

BBB	Blood–brain barrier
CNS	Central nervous system
ED2	CD163
HRP	Horseradish peroxidase
qRT-PCR	Quantitative reverse transcriptase polymerase chain reaction
VEGF	Vascular endothelial growth factor
VEGFR	Vascular endothelial growth factor receptor
18S	18S ribosomal RNA
