# Supplementary material for: Increased Expression of Vascular Endothelial Growth Factor-D Following Brain Injury
Source: Int J Mol Sci. 2019 Mar 30;20(7):1594. doi: 10.3390/ijms20071594 (PMC6479775; doi:10.3390/ijms20071594)
Supplement: Supplementary file 1 [file ijms-20-01594-s001.pdf]

VEGF-D Protein Expression

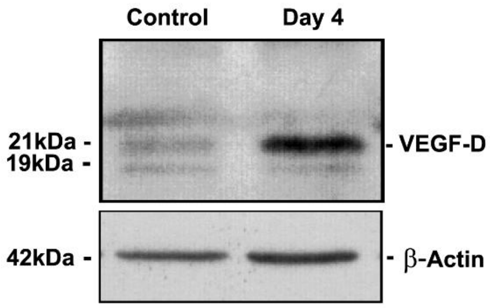

Figure S1 Image from a representative western blot showing increased VEGF-D expression (21 kDa) in the brain of a day 4 cold-injured rat as compared to the control rat brain. Immuno-blotting was performed as described previously [30].
